# Cardiac Tamponade: An Unusual Cause of a Severe Headache with Normal Blood Pressure

**DOI:** 10.7759/cureus.7004

**Published:** 2020-02-15

**Authors:** Phool Iqbal, Safna Farsana Akkam Veettil, Arwa Alsaud, Nagham Sadik, Sania Razzaq

**Affiliations:** 1 Internal Medicine, Hamad General Hospital, Doha, QAT; 2 Radiology, Hamad General Hospital, Doha, QAT

**Keywords:** cardiac tamponade, normal blood pressure, headache

## Abstract

Cardiac tamponade is a life-threatening condition characterized by slow or rapid compression of the heart due to the accumulation of fluid in the pericardial space and rarely presents mainly as a headache.

We report an unusual presentation of cardiac tamponade associated mainly with severe headache over three days and mild shortness of breath in a 60-year-old male not known to have any previous heart disease. Immediate computed tomography (CT) scan of the head ruled out intracranial hemorrhage. A chest x-ray showed cardiomegaly, and further echocardiography revealed a large pericardial effusion on a transthoracic echocardiogram compromising the right ventricular output. Urgent pericardiocentesis was performed and removed 800 ml of hemorrhagic fluid that led to a complete resolution of his severe headache.

## Introduction

Cardiac tamponade is a clinical syndrome caused by the accumulation of fluid, gas, pus, or blood in the pericardial space, resulting in reduced diastolic ventricular filling and subsequent hemodynamic compromise [1]. The condition is a medical emergency that can lead to cardiac arrest and death if not treated in a timely manner [2].

Headache as the main presentation of a potentially fatal condition like cardiac tamponade without any underlying secondary cause is quite an uncommon finding in the literature. Herein, we are reporting the case of a 60-year-old male with an unusual presentation of cardiac tamponade.

## Case presentation

A 60-year-old morbidly obese gentleman, a chronic smoker and alcohol drinker with a medical history significant for uncontrolled hypertension, type 2 diabetes mellitus with microvascular complications, stroke without residual weakness, and obstructive sleep apnea, presented to the emergency department with a three-day history of progressive severe headache. He described it as the worst headache of his life, involving the whole cranium and associated with mild blurriness of vision but without photophobia or any other focal neurological deficit. 

He had cervical and lumbar spine decompression surgery and a renal transplant 11 years earlier, as well as stable renal graft function on immunosuppressant medications (prednisolone, tacrolimus, and mycophenolate).

On physical examination, the patient was having mild distress in a sitting position but was hemodynamically stable, afebrile with normal blood pressure, a pulse rate of 98/min, and a respiratory rate of 24/min on 3L/min nasal cannula maintaining oxygen saturation of 97%. His body mass index (BMI) was 42 kg/m^2^. He had mild bilateral pedal edema. We were unable to comment on jugular venous pulsations and heart sounds due to his thick short neck and chest wall, respectively. Neurological examination was unremarkable for any focal motor or sensory deficit and cranial nerve examination was normal. Fundoscopic examination of the optic disk was unremarkable for any papilledema.

**Figure 1:**
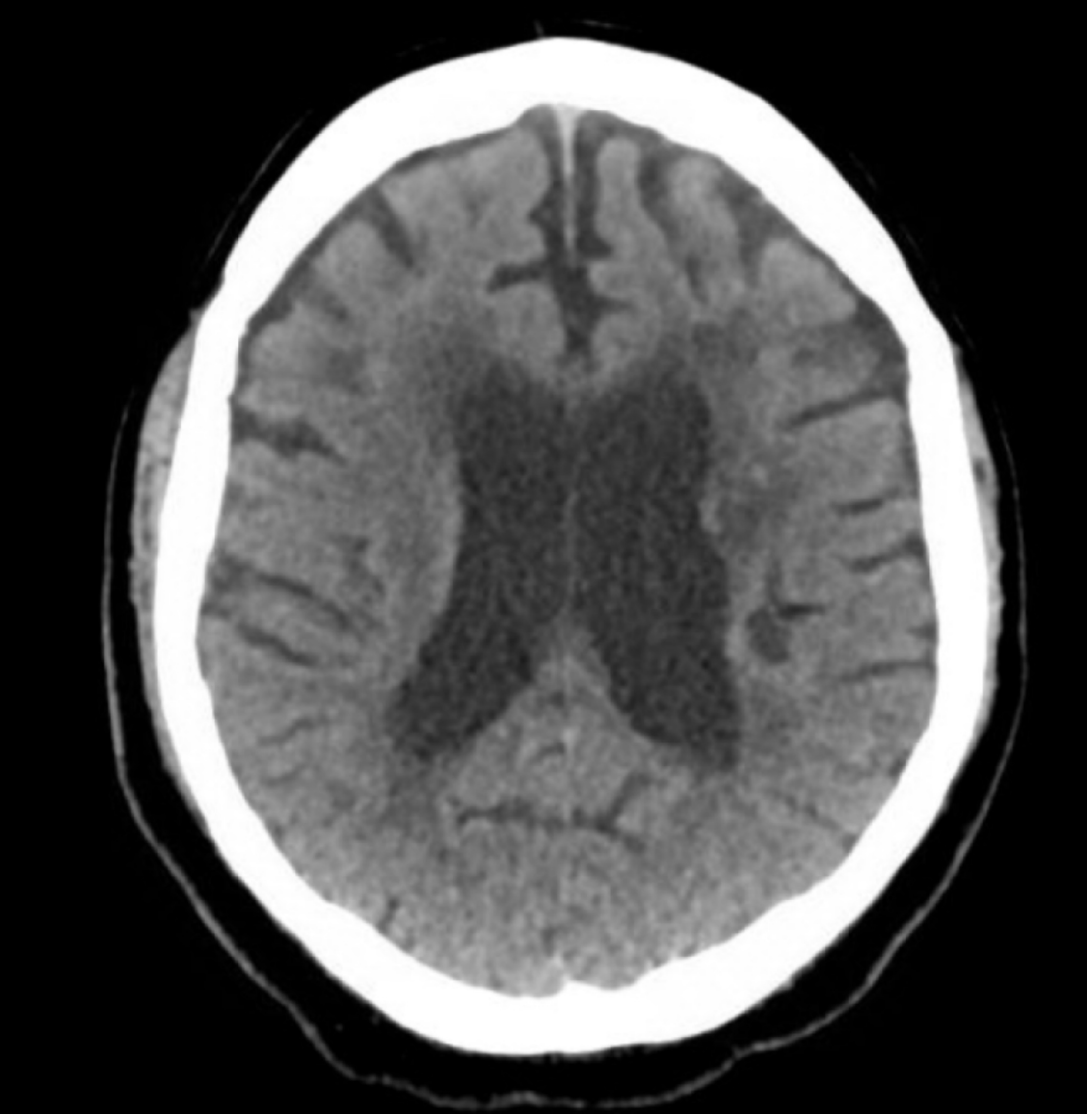
Computed tomography scan brain unremarkable for subarachnoid hemorrhage or any other acute insult

**Figure 2:**
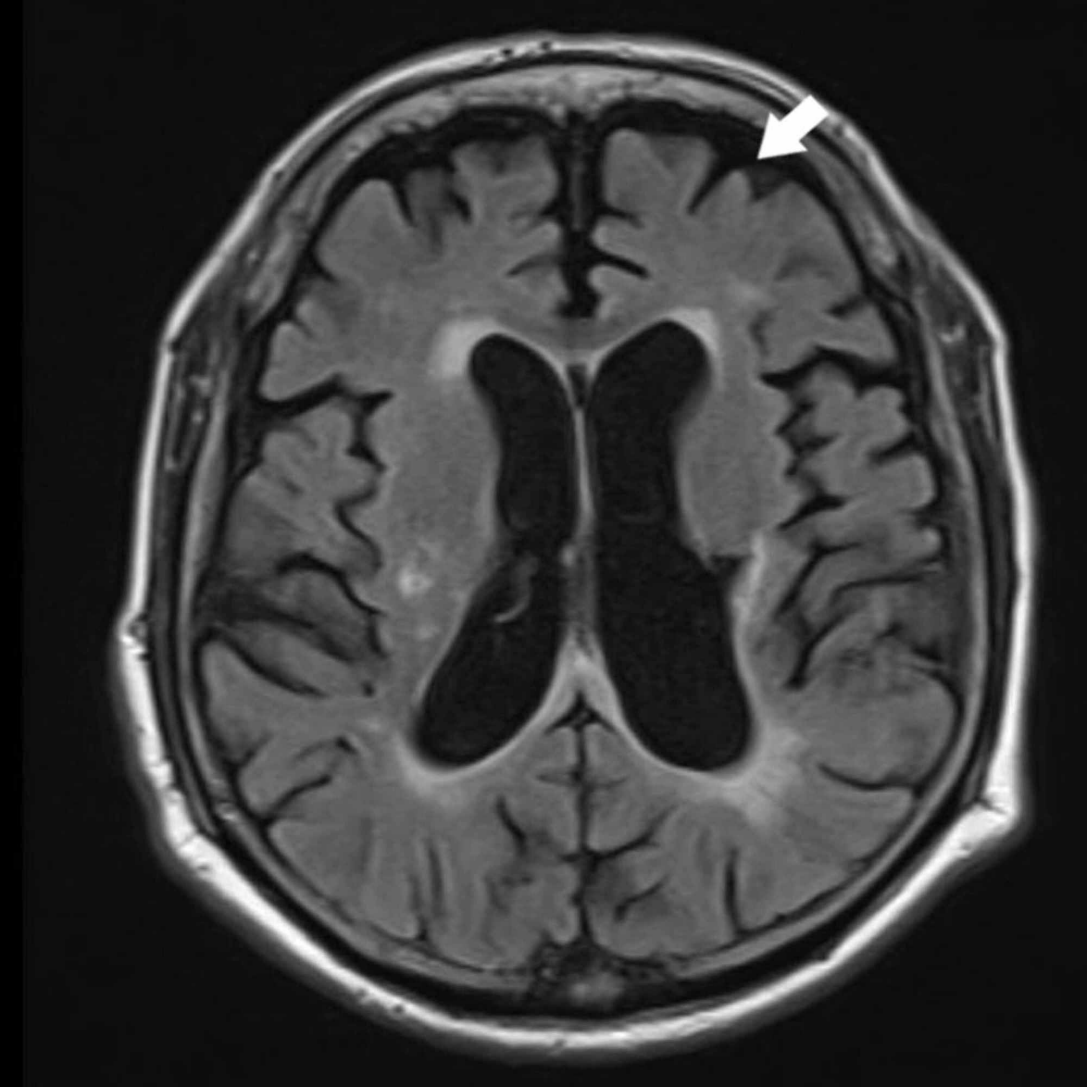
Magnetic resonance imaging (MRI) of the brain The arrow points towards sulcal widening and gyri atrophy correlating with generalized brain volume loss and atrophy

An urgent computed tomography (CT) scan brain ruled out subarachnoid hemorrhage as shown in Figure 1. Magnetic resonance imaging (MRI) of the brain with contrast showed generalized volume loss of the brain as shown in Figure 2. Lumbar puncture was difficult to perform due to the history of metallic fixators in the lumbar spine region.

**Figure 3:**
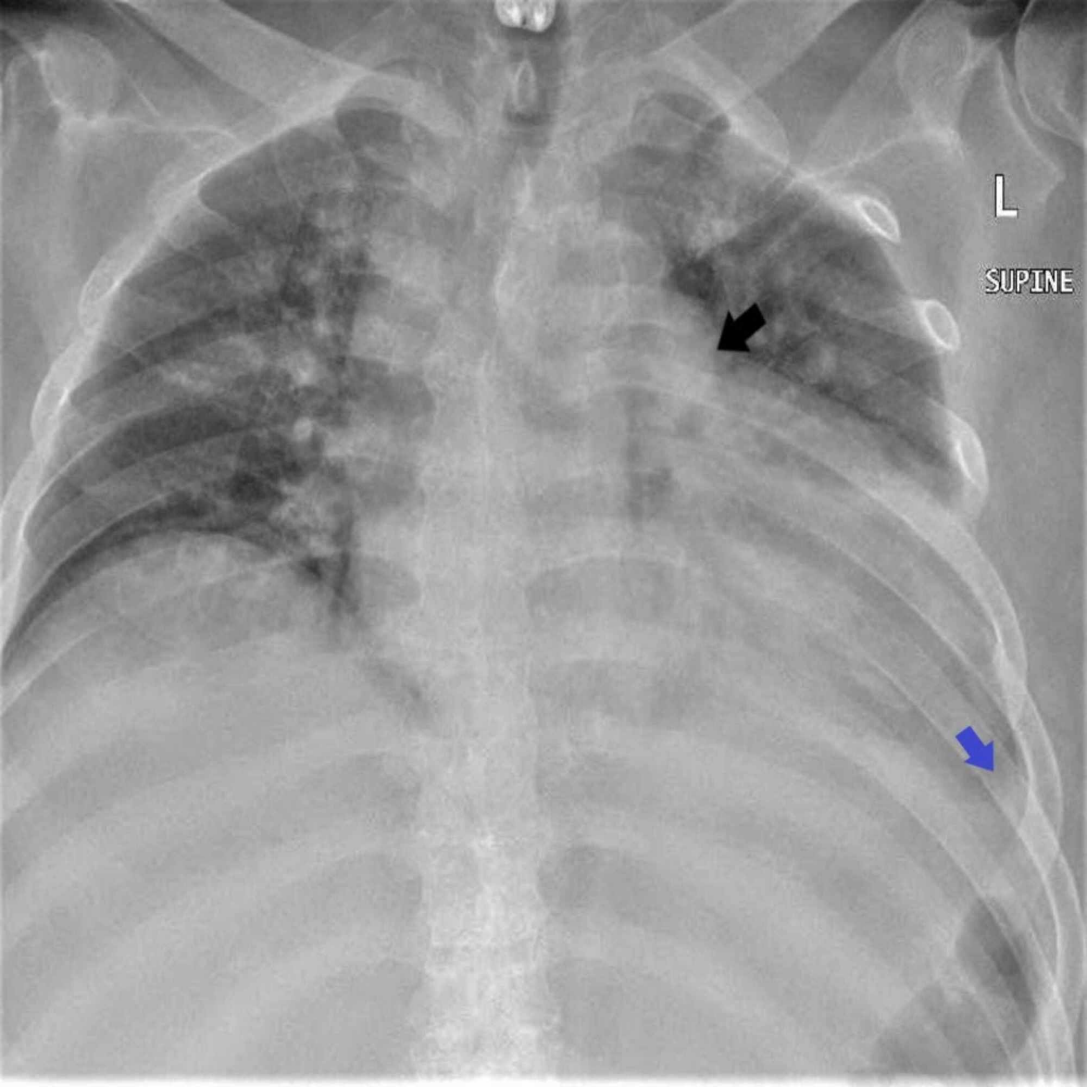
X-ray revealing a large pericardial effusion with features of tamponade The black arrow points towards the increased cardiac silhouette suggestive of pericardial effusion; the blue arrow shows a left blunted costophrenic angle due to pleural effusion.

An electrocardiogram (ECG) showed low voltage sinus rhythm and no beat-to-beat variation. Chest x-ray showed mild left pleural effusion and cardiomegaly as seen in Figure 3. Echocardiography was performed which revealed a large pericardial effusion with features of tamponade that included a large circumferential pericardial effusion with respiratory variation greater than 25% and right ventricular compression during systole and diastole.

**Figure 4:**
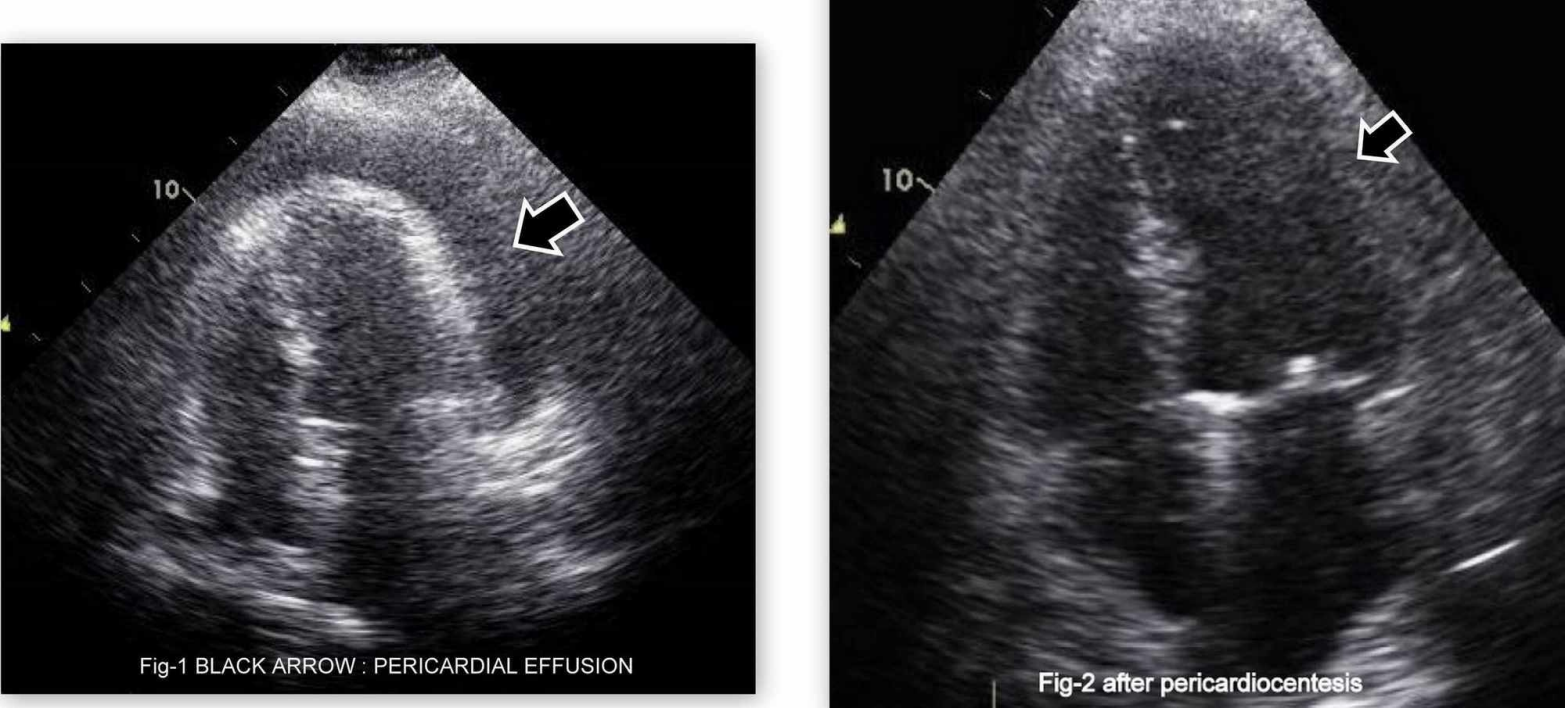
Before (left side) and after (right side) pericardiocentesis echocardiography of the patient

The patient was taken to the coronary care unit and underwent emergent pericardial drainage as described in Figure 4. Approximately 800 mL of hemorrhagic pericardial fluid was drained. The fluid analysis showed 750 /uL white blood cells with mainly 74% neutrophilic, 1% lymphocytes and 50,750 RBC/uL concluding it as a hemorrhagic pericardial effusion. Acid-fast bacilli (AFB) smear, tuberculosis (TB) polymerase chain reaction (PCR), fluid culture, and viral markers were negative from the pericardial fluid analysis. Vasculitis screening with antinuclear antibody (ANA) and antineutrophil cytoplasmic antibody (ANCA) was negative as well. The septic screen was also sent for the patient and his blood cultures did not grow any microorganism.

The patient’s headache was relieved after pericardiocentesis. His headache did not recur during his remaining length of hospital stay. However, his blood pressure started to shoot up to 180 - 220 mmHg systolic over 90 - 120 mmHg diastolic for which we resumed his home antihypertensive medications and it stabilized.

**Figure 5:**
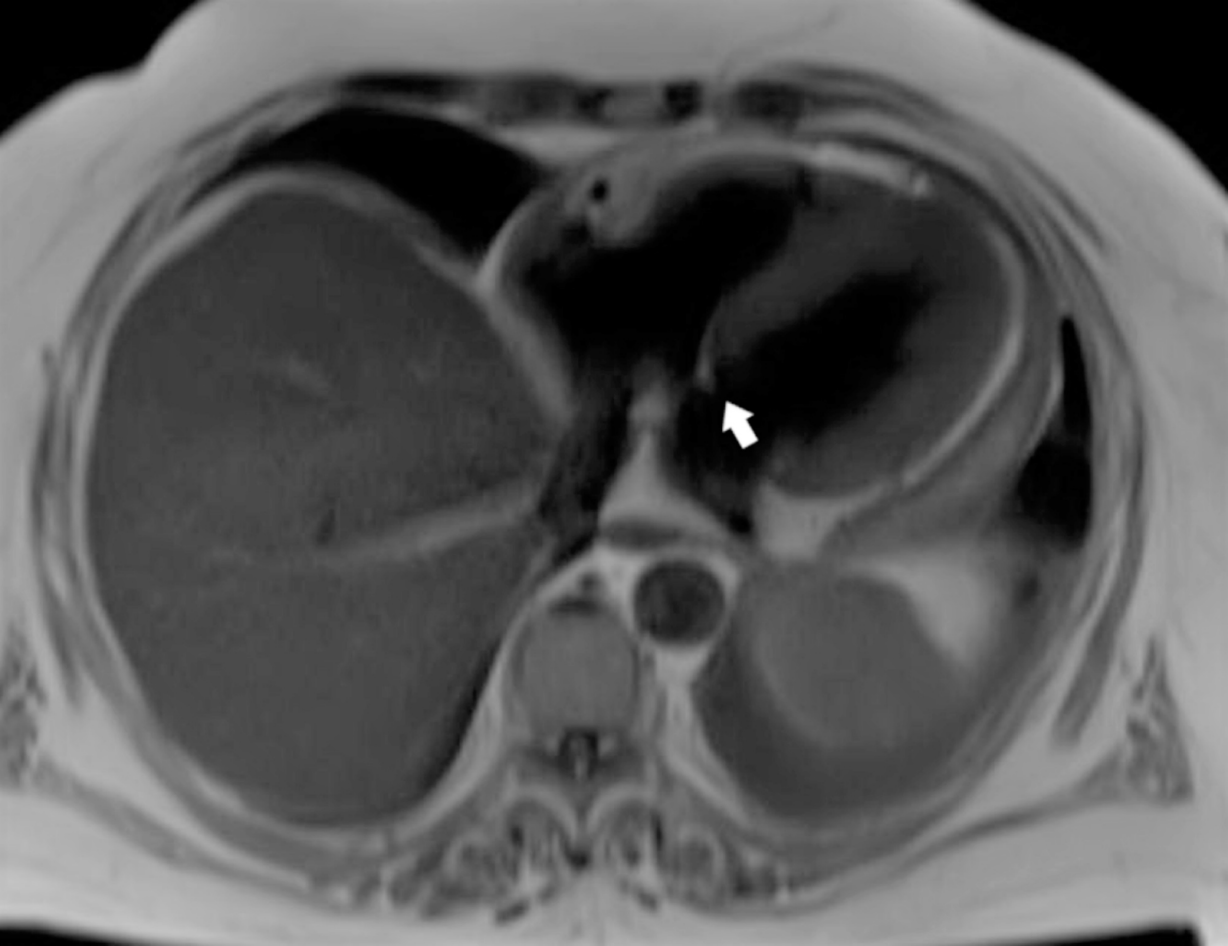
Cardiac magnetic resonance imaging (MRI) showing basal septal mid-wall enhancement of non-ischemic type consistent with myocarditis or cardiomyopathy

A positron emission tomography/computed tomography (PET CT) scan did not detect any malignant focus with enhanced uptake. An MRI scan of the heart showed basal septal mid-wall myocardial enhancement of non-ischemic pattern, likely due to myocarditis or cardiomyopathy as shown in Figure 5.

After ruling out secondary causes of hemorrhagic pericardial effusion, such as malignancy, autoimmune diseases, and TB, our patient was labeled as idiopathic myocarditis leading to chronic pericardial effusion based on MRI scan of the heart. We observed the patient in the hospital with a regular echocardiogram over 10 days. He was asymptomatic and discharged with regular follow-up in the cardiology clinic. Cardiologist outpatient follow-ups with repeated echocardiogram studies for a further five months did not show any fluid reaccumulation. The patient was followed for one year after discharge where he remained clinically stable.

## Discussion

The heart is enclosed in the pericardium which is a double-layered sac. Pericardial fluid is a lubricating substance in between the two layers of the pericardium and is approximately 50 ml in quantity. Its main function is to reduce the friction between the pericardial layers and provide antimicrobial properties [3].

The hemodynamics of the heart in cardiac tamponade is disrupted. It occurs when the pressure inside the heart chambers is insufficient to overcome the increased interpericardial pressure during diastole. The heart chambers also become smaller in size due to external pericardial pressure during the cardiac cycle, leading to a decreased venous return to the right side of the heart. Consequently, it compromises cardiac output leading to hypotension. However, tachycardia is seen as one of the early compensatory response mechanisms to maintain cardiac output [3-5]. Signs and symptoms of cardiac tamponade depend on the rate of accumulation, i.e., acute versus subacute or chronic, pericardial stretch, and ventricular compliance [3-4].

A classic presentation of cardiac tamponade includes Beck’s triad which is hypotension, increased jugular venous pulse, and muffled heart sounds, while chest pain, dyspnea, tachycardia, pulsus paradoxus, and cardiovascular collapse can also occur. In acute settings, the patient can deteriorate within minutes even with minimal increase in pericardial fluid up to 150 ml [3-5].

In subacute or chronic settings, the stretch of the pericardium is accommodative to slow rising interpericardial fluid. Therefore, it can retain even more than 1,000 ml over days to months, and the patient may still be asymptomatic as long as diastolic pressure of the heart is more than pericardial pressure [3]. Once, the diastolic pressure is no longer sufficient enough to overcome the rising pericardial pressure, the patient becomes symptomatic and can present with fatigability, mild dyspnea, peripheral edema, and chest discomfort. Rare chronic cases can have a circulatory collapse [3-5].

There is a chronic compression triad similar to Beck's triad, and it is accompanied by high venous pressure, abdominal ascites, and muffled heart sounds [6].

Our patient did not present with typical features of cardiac tamponade and came primarily with the symptom of headache, which is rare to be seen in such life-threatening conditions with relatively normal blood pressure.

Headache due to cardiac tamponade can be explained by the inability of cerebral veins to drain into the superior vena cava and then into the right atrium due to external compression by massive pericardial effusion. As cerebral vessels are pain-sensitive structures, this leads to stretch on the cerebral vessels that trigger pain sensation and traction type of headache [7-8].

Headache in association with cardiac tamponade has been reported in few case reports, but there were underlying diseases, such as dengue fever, lupus nephritis, and superior vena cava syndrome, which explained the headache in those patients [9-11].

Our patient was known to have uncontrolled blood pressure and was on four different types of antihypertensive medications. However, due to the cardiac tamponade, he presented with normal blood pressure which created a dilemma to diagnose such scenarios. This emphasizes the importance of history taking and background in such patients.

He had mild shortness of breath, his heart sounds were difficult to detect due to thick chest walI, and jugular venous pulsations could not be appreciated due to a short neck. Therefore, the decision of echocardiography was made after his chest x-ray reported cardiomegaly and suspected pericardial effusion. This led to the diagnosis of massive pericardial effusion leading to tamponade. After urgent pericardiocentesis, the patient's headache and shortness of breath were relieved and his blood pressure started to shoot up which required us to resume his antihypertensive medication.

The pericardial fluid analysis revealed hemorrhagic pericardial effusion, and the patient underwent extensive investigations that ruled out secondary causes like tuberculosis, malignancy, and autoimmune diseases. Thus, it was determined to be a case of idiopathic myocarditis on basis of cardiac MRI, complicated by massive chronic hemorrhagic pericardial effusion and cardiac tamponade. Idiopathic myocarditis is a common cause of pericardial effusion found in the literature. We did a close follow-up for one year and the patient remained asymptomatic without any reaccumulation of pericardial effusion on repeat echocardiography as an outpatient. However, if there should be a recurrence of pericardial effusion and no other cause can be established from clinical and laboratory investigations, then a biopsy can help in such clinical scenarios [12]. Our patient did not require it.

Echocardiography is the best modality to look for pericardial effusion and is used as a tool in follow-up [2-3]. Our patient was followed with serial transthoracic echocardiography and he remained stable throughout our follow-up.

## Conclusions

Cardiac tamponade is a life-threatening condition that can lead to cardiac arrest and death if not promptly managed. Our patient mainly presented with a severe intractable headache associated with this fatal condition which was relieved immediately after pericardiocentesis. Our main goal is to highlight the importance of history taking, physical examination, and approach towards this atypical presentation associated with cardiac tamponade and its urgent management for better patient care. As per the literature review, and to our knowledge, headache as the main presenting symptom of cardiac tamponade due to idiopathic myocarditis has not been reported.
